# Lemierre Syndrome: An Uncommon Cause of Septic Embolism

**DOI:** 10.1590/0037-8682-0363-2021

**Published:** 2021-08-20

**Authors:** Antônio Carlos Portugal Gomes, Eduardo Mattar, Edson Marchiori

**Affiliations:** 1 Medimagem/BP Medicina Diagnóstica. São Paulo, SP, Brasil.; 2 Telemedimagem - Hospital Alvorada Moema, São Paulo, SP, Brasil.; 3 Universidade Federal do Rio de Janeiro, Rio de Janeiro, RJ, Brasil.

An 18-year-old female patient presented to the emergency department with complaints of high fever (40.2°C), chills, and sore throat. She also had chest pain, dyspnea, and tachypnea (respiratory rate:30 breaths/min; 80% oxygen saturation). Physical examination revealed edematous and congested oropharyngeal mucosa, along with acute tonsillitis and right-sided neck edema. Laboratory tests revealed leukocytosis (leukocyte count, 18,000/mm^3^) and elevated levels of C-reactive protein (26.3 mg/L). Blood culture was positive for anaerobic *Fusobacterium necrophorum*.

Chest computed tomography (CT) revealed multiple bilateral nodules, most of which were cavitated (Figure 1A, B). Contrast-enhanced CT of the head and neck revealed a hypoechoic thrombus filling the right internal jugular vein (Figure 1C, D). Color Doppler imaging revealed a thrombus and the absence of detectable intraluminal flow (Figure 1E). The patient was diagnosed with Lemierre syndrome secondary to oropharyngeal infection. Her symptoms improved with antibioticotherapy, and she was discharged in a stable condition.

**FIGURE 1:**
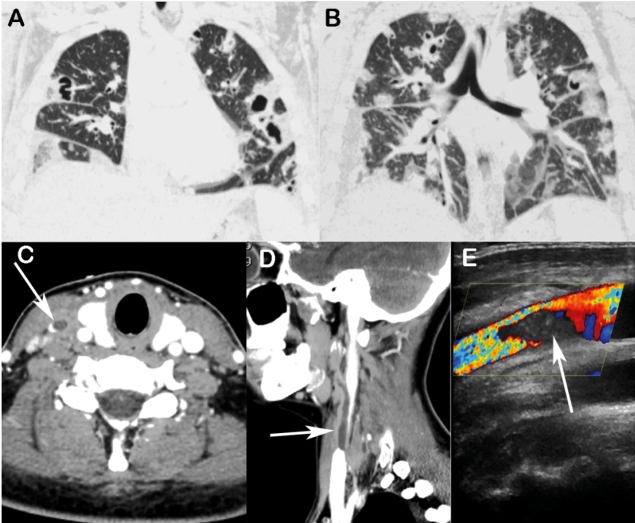
Chest computed tomography (CT) with coronal reconstruction **(A and B)** demonstrating multiple bilateral pulmonary nodules, most of which were cavitated and associated with areas of consolidation. Right pleural effusion should also be noted. Contrast-enhanced CT of the neck region with axial **(C)** and sagittal **(D)** reconstruction showing a hypoechoic thrombus in the right internal jugular vein (arrows). Color Doppler imaging (E) also demonstrating the thrombus (arrow).

Lemierre syndrome is defined as septic thrombophlebitis of the internal jugular vein that becomes a source of septic emboli in the setting of throat infection. The most commonly involved organism was *F.necrophorum*. When not recognized and treated aggressively, primarily with broad-spectrum antibiotics, this syndrome causes significant morbidity and mortality^1^ ^,^ ^2^.

## References

[1] Jafri FN, Shulman J, Gómez-Márquez JC, Lazarus M, Ginsburg DM (2018). Sore Throat, Fever, Septic Emboli, and Acute Respiratory Distress Syndrome: A Case of Lemierre Syndrome. Case Rep Emerg Med.

[2] Jácome F, Dias-Neto M (2021). Septic Pulmonary Embolism Secondary to Lemierre Syndrome. Eur J Vasc Endovasc Surg.

